# Trends of Private Drugs' Sales and Costs Incurred by Patients on Anti-tuberculosis Drugs in Selected Districts of Jharkhand (2022): Results From Sub-national TB-Free Certification

**DOI:** 10.7759/cureus.47296

**Published:** 2023-10-18

**Authors:** Ratnesh Sinha, Rishabh K Rana, Anit Kujur, G Jahnavi, Mithilesh Kumar, Vinayagamoorthy Venugopal, Neha Priya, Manisha Kujur, Ravi Ranjan Jha, Rajan Barnwal, Nikhil Nishant, Nisha Murmu, Rajeev Pathak, Anupama T, Ranjit Prasad, Rakesh Dayal, Bhavesh Modi, Anil J Purty, Sharath BN, Dina Nair, Dewesh Kumar

**Affiliations:** 1 Department of Community Medicine, Manipal Tata Medical College, Manipal Academy of Higher Education, Jamshedpur, IND; 2 Department of Preventive and Social Medicine/Community Medicine, Shaheed Nirmal Mahto Medical College and Hospital (Erstwhile Patliputra Medical College), Dhanbad, IND; 3 Department of Community Medicine, Rajendra Institute of Medical Sciences, Ranchi, IND; 4 Department of Community Medicine and Family Medicine, All India Institute of Medical Sciences, Deoghar, Deoghar, IND; 5 Department of Community and Family Medicine, All India Institute of Medical Sciences, Deoghar, Deoghar, IND; 6 Department of Preventive Medicine, Rajendra Institute of Medical Sciences, Ranchi, IND; 7 Department of Community Medicine, Shaheed Nirmal Mahto Medical College and Hospital (Erstwhile Patliputra Medical College), Dhanbad, IND; 8 Department of Community Medicine, Mahatma Gandhi Memorial Medical College and Hospital, Jamshedpur, IND; 9 Department of Community Medicine, Medinirai Medical College, Palamu, IND; 10 Department of Preventive Medicine, All India Institute of Medical Sciences, Bhubaneswar, Bhubaneswar, IND; 11 NTEP Technical Support Network, World Health Organization, Ranchi, IND; 12 State TB Cell, Health Services, Government of Jharkhand, Ranchi, IND; 13 Department of Community and Family Medicine, All India Institute of Medical Sciences, Rajkot, Rajkot, IND; 14 Department of Community Medicine, Pondicherry Institute of Medical Sciences, Pondicherry, IND; 15 Department of Community Medicine, Employees' State Insurance Corporation (ESIC) Medical College and Post Graduate Institute of Medical Science & Research (PGIMSR), Bengaluru, IND; 16 Department of Clinical Research, Indian Council of Medical Research-National Institute for Research in Tuberculosis (ICMR-NIRT), Chennai, IND; 17 Department of Community Medicine/Preventive and Social Medicine, Rajendra Institute of Medical Sciences, Ranchi, IND

**Keywords:** att drugs, catastrophic expenditure, direct cost, out-of-pocket expenditure, tuberculosis

## Abstract

Background: The government of India is committed to eliminating tuberculosis (TB) by 2025 under the National Tuberculosis Elimination Programme which provides free investigations and treatment as well as incentives for nutritional support during their treatment course. Many TB patients prefer to seek treatment from the private sector which sometimes leads to financial constraints for the patients. Our study aims to find the burden of TB patients in the private sector and the expenses borne by them for their treatment.

Methodology: Sales data of rifampicin-containing formulation drug consumption in the private sector of six districts of Jharkhand was collected from Clearing and Forwarding agencies. Based on the drug sales data, the total incurring costs of the drugs, total number of patients, and cost per patient seeking treatment from the private sector were calculated for the year 2015-2021. ANOVA and the post hoc test (Tukey honestly significant difference (HSD)) were applied for analysis.

Results: There was a marked difference amongst all the districts in relation to all the variables namely total costs, cost per patient, and total private patients seeking treatment from the private sector which was statistically significant (p < 0.001). East Singhbhum had the highest out-of-pocket expense and private patients as compared to all six districts. Lohardaga showed the sharpest decline in total private patients from 2015 to 2021. The average cost borne by private patients in 2015 was INR 1821 (95% CI 1086 - 2556) which decreased to INR 1033 (95% CI 507 - 1559) in 2021.

Conclusion: From the study, it was concluded that the purchase of medicines for TB treatment from the private sector is one of the essential elements in out-of-pocket expenditure (OOPE) borne by TB patients. Hence, newer initiatives should be explored to foresee the future OOPE borne by the patients and decrease OOPE-induced poverty.

## Introduction

Tuberculosis (TB) has burdened humans not only due to its effects as a medical condition but also its impact as a social and economic tragedy [1]. There has never been a disease in human history that has caused such suffering in terms of morbidity and mortality. Historically, millions of people have been killed by several other diseases like smallpox and plague, but their reign has been short-lived [2]. India has the highest estimated burden of tuberculosis infection (TBI) globally, approximately 350-400 million. Out of these 2.6 million people (1.8-3.6 million) are estimated to develop TB disease annually [3]. TB causes an annual loss of approximately 100 million dollars to the Indian economy. World Health Organization (WHO) TB India 2019 statistics indicate that India loses approximately USD 32 billion annually to the disease. TB mortality is expected to cost US$32 billion each year over the next 30 years [4]. There are also several indirect costs in the form of workflow disruption, reduction in productivity, loss of employment, and the replacement and retraining of workers [5,6].

Through the National TB Elimination Programme (NTEP), India aims to eliminate TB by the year 2025, five years ahead of the global target. To achieve this the “National Strategic Plan (NSP) of 2017-2025” has been launched to mark a departure from previous plans with a higher financial commitment, increased private sector engagement, and focus on a more holistic approach. It lays emphasis on providing financial and nutritional assistance, free diagnosis, and treatment to all TB patients by ‘going where the patient goes.’

In the fight against TB, the Central TB Division (CTD), Ministry of Health Family Welfare (MoHFW), Government of India (GoI) is adopting newer innovative strategies. The CTD initiated a Pan India project in 2020 for “TB-free status” in all the districts or states focusing on an 80% reduction in overall TB incidence from the baseline (numbers in 2015) for achieving a “TB-free status”. Since it may take years, an interim recognition in the form of awards and certifications for the progress towards a TB-free status under the categories bronze, silver, and gold for 20%, 40%, and 60% reduction, respectively. This is to motivate, inspire and instil competitiveness in the mindset of the various stakeholders and those who are working towards the elimination of TB [7]. Despite the provision of free diagnosis and treatment by the Government under the NTEP, the cost incurred by patients and their families is quite considerable, triggering a spiral towards deeper poverty [8,9].

In developing countries, such as India, due to low health budgets and a larger presence of the private sector force patients to incur out-of-pocket (OOP) expenses to meet their health demands [10]. OOP is the direct payment borne by any individuals at the time for use of health services and nonmedical payments [11]. This problem was also recognized by the WHO in their End TB Strategy, which includes a target of no TB-affected family facing catastrophic costs because of TB by 2020 [12]. The stagnation in the effectiveness of major health programs due to the COVID-19 pandemic has also contributed to this [13].

Despite several government initiatives, the private healthcare sector accounts for 74% of primary TB treatment-seeking and 54% of TB medicine consumption [14]. Data on the cost incurred by patients towards TB care and its impact including the economic burden are not routinely collected. There have been minimal studies done on the direct costs incurred on drugs by patients. The state-wise distribution number of patients seeking private healthcare for TB is also obscure. As part of the sub-national TB free certification process 2022 in select districts of the state of Jharkhand, we determined (a) the quantity of anti-TB drugs sold in the private sector, (b) the estimated number of TB patients in the private sector, and (c) the cost incurred by the TB patients for the purchase of drugs. This study also highlights the trends of costs incurred on drugs being bought in private in the last five years in six different districts of the state.

## Materials and methods

NTEP has strategized Sub-National Certification (SNC) of various qualitative grades by verification of the claims made by districts in their respective states of India. This verification was done by public health experts through a community-based survey and review of the programmatic data available in the existing NI-KSHAY portal and anti-TB drug utilization. The selection of these six districts (Khunti, Deoghar, Pakur, East Singhbhum, West Singhbhum, and Lohardaga) of Jharkhand was based on the nomination by the State authorities for SNC as these are districts that have claimed a bronze medal i.e.,>20% decrease in the incidence of TB.

For the current study, we utilized the secondary data of drug consumption from the private sector of Jharkhand state collected during the verification process for further analysis. The verification process was done from February 2022 to March 2022 in six districts of Jharkhand. The sales data of rifampicin-containing formulations were collected from Clearing and Forwarding (C & F) agencies and the drug controller office. All types of formulations from fixed-dose combinations to separate tablets and paediatric to adult doses were included. After the data collection from the field, Nikshay portal, and drug controller office of state, a desk review of the district-level drug consumption was done to meet the objectives of the study.

Qualitative data (sales coverage, treatment duration, extent of prescription of products for TB treatment, treatment covered by each unit of product) obtained by Nominal Group technique (NGT) and Key Informant Interviews (KII) by interviewing chemists and private medical practitioners from clinics and nursing homes/hospitals were also considered. There were issues with the private sector drug sale data which also challenged its validity including the incomplete nature of records and its maintenance. Although some rifampicin-containing drugs are also used in other conditions which have been obtained through NGT, consideration has been given to them during our calculations. Migratory TB patients seeking treatment from other districts have been taken into account for the estimation of drug sales in the study.

Data analysis 

Drug sales data were collected, and different variables were calculated as per the equation explained below.

a) Ti- For a given drug i, consumed in the private sector, Ti represents the number of treatment months for each unit sold needed to treat TB. The private drug sale data along with the retail price of six districts for 2015-2021 year were compiled in a Microsoft Excel 2017 sheet and further analysis of the data was done in Jamovi.

b) Out-of-pocket expenditure (OOPE) of purchase of ATT drugs from the private sector

Cost of each drug = no of pack sold per year * cost of each pack

Total OOPE = ∑Cost of each drug

c) Number of private patient's months

Number of private patient's months for a particular drug = no of packs sold * Ti value of that drug

Total number of private patient’s months = ∑No of private patients months for each drug

d) Number of private patients in a year

Number of private patients = (Total no of patients taking medicine from the private sector in a year)/6

e) OOPE on ATT drugs purchase from the private sector per patient

OOPE per patient = Total OOPE/ Total no of patients in the given year

The data collected after analysis has been expressed after univariate analysis in mean, standard deviation, and its 95% confidence interval. Statistical test ANOVA and post hoc test Tukey honestly significant difference (HSD) method were applied to test the significance and note any statistical difference between the groups. P<0.05 was considered statistically significant.

Ethics approval

This study involves human participants and was approved by the Institutional Ethics Committee, National Institute of Epidemiology, Chennai, ID: NIRT-IEC No: 2021 049]”, dated 1st March 2022.

## Results

The costs of drugs were calculated from the data gathered at the state level from the state controller of Jharkhand. They were also adjusted as per the information provided by the private practitioners, chemists, and district officials. The participating districts in the sub-national certification process in 2022 from Jharkhand were Khunti, Deoghar, Pakur, East Singhbhum, West Singhbhum and Lohardaga.

**Figure 1:**
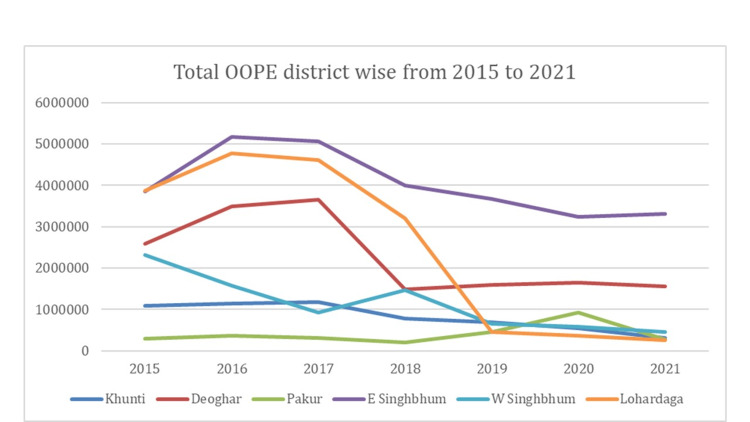
Total OOPE in INR year-wise in all districts from 2015 to 2021 OOPE: Out-of-pocket expenditure

Our study estimated yearly sales of anti-TB drugs from 2015 to 2021in six districts which varied from approximately INR 4 lakhs to 40 lakhs. The total drug costs over the study period have decreased in almost all studied districts (Figure 1) but the sharpest decline was observed in Lohardaga from 2015 to 2021.

**Figure 2:**
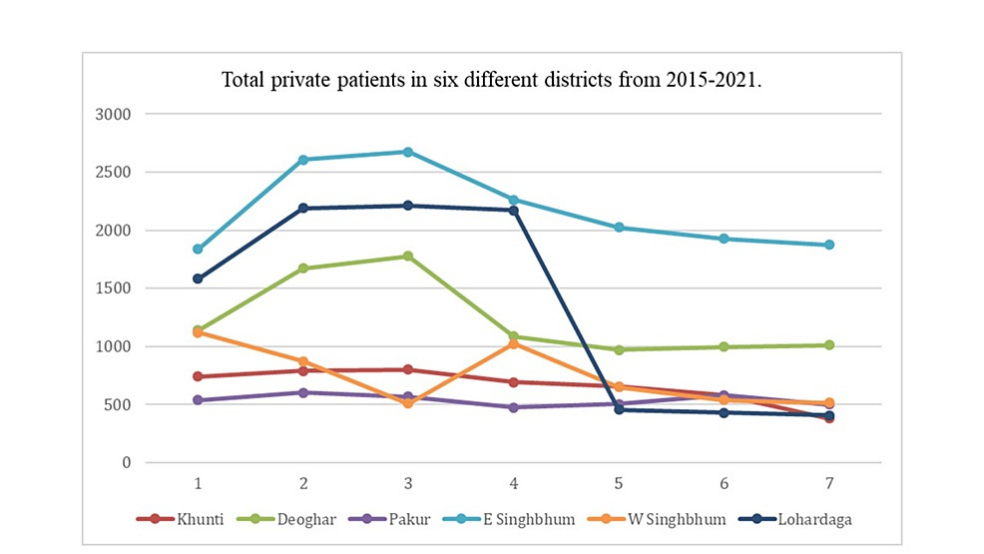
Total private patients in six different districts from 2015 to 2021

The number of private patients has also steadily decreased over the study period in all the districts except East Singhbhum which had a marginal increase in 2021 in comparison to the base year i.e., 2015 (Figure 2).

The average cost borne by private patients for their treatment duration ranged from 553 INR (Pakur) to 2441 INR (Lohardaga) approximately in 2015 which has decreased over the seven years in almost all districts (Table 1).

**Table 1:** Average drug costs borne by private patients in the six districts from 2015 to 2021

OOPE per Patient	2015	2016	2017	2018	2019	2020	2021
Khunti	1483.9	1446.4	1478.6	1135.2	1053.1	947.2	806.0
Deoghar	2276.9	2088.0	2056.7	1361.0	1643.8	1653.4	1535.9
Pakur	553.16	600.80	540.69	446.96	906.42	1591.15	562.33
East Singhbhum	2092.9	1987.0	1895.4	1768.1	1817.7	1685.2	1770.1
West Singhbhum	2076.3	1798.9	1840.2	1438.2	1000.1	1078.2	900.3
Lohardaga	2441.8	2185.8	2085.3	1479.2	983.4	865.9	622.4
Mean	1821	1684	1649	1302	1289	1354	1033
95% CI	1086 – 2556	1064 – 2305	1036 - 2263	896 - 1708	913 – 1665	891 – 1817	507 - 1559
IQR	599	528	447	277	528	697	709

The total costs, total private patients, and cost per patient seeking treatment from the private sector were calculated for years 2015-2021. There was a marked difference amongst all the districts and the difference in all variables was statistically significant (p<0.001) after applying the ANOVA test (Table 2).

**Table 2:** District-wise comparison of mean expenditure in INR on drugs and number of patients over the period 2015-2021

		Mean in INR	Std. Error (INR)	95% Confidence Interval for Mean		p-value
				Lower Bound (INR)	Upper Bound (INR)	
Total out-of-pocket expenditure in INR	Khunti	821198.14	126190.47	512421.18	1129975.1	<0.001
	Deoghar	2287055.35	362248.09	1400666.2	3173444.5	
	Pakur	405438.67	90398.5	184241.5	626635.84	
	East Singhbhum	4048618.10	295922.66	3324521.43	4772714.76	
	West Singhbhum	1142662.72	255830.29	516668.55	1768656.88	
	Lohardaga	2507721.79	784705.2	587617.35	4427826.24	
Total private patients	Khunti	661.67	55.35	526.24	797.11	<0.001
	Deoghar	1235.7	128.84	920.43	1550.97	
	Pakur	538.29	17.7	494.97	581.6	
	East Singhbhum	2172.43	131.91	1849.66	2495.2	
	West Singhbhum	747.13	96.88	510.07	984.18	
	Lohardaga	1349.99	335.18	529.82	2170.15	
Out-of-expenditure in INR per patient	Khunti	1192.91	105.05	935.86	1449.97	<0.001
	Deoghar	1802.24	127.67	1489.84	2114.64	
	Pakur	743.07	151.46	372.45	1113.69	
	East Singhbhum	1859.49	53.56	1728.42	1990.55	
	West Singhbhum	1447.46	176.55	1015.45	1879.46	
	Lohardaga	1523.4	273.24	854.82	2191.98	

As per cumulative data of 2015-2021, the highest expenditure on drugs was seen in East Singhbhum, followed by Lohardaga, and the least was observed in Pakur. In the analysis of cost per patient, East Singhbhum topped the list, followed by Deoghar with Pakur at the bottom. Like all other parameters, East Singhbhum has a maximum number of private patients, followed by Lohardaga and Pakur has the least number of patients seeking treatment from the private sector in the last seven years. After application of the post-hoc test (Tukey HSD method), it was seen that the difference exists in all the variables namely total costs, total private patients and cost per patient seeking treatment from the private sector among the diverse groups (Table 3).

**Table 3:** Comparison of the difference of total out-of-pocket expenditure, number of patients, and out-of-pocket expenditure per patient among the six districts

		Deoghar	East Singhbhum	Khunti	Lohardaga	Pakur	West Singhbhum
Tukey Post-Hoc Test – Total Out-of-Pocket Expenditure							
Deoghar	p-value	—	0.034	0.115	0.999	0.026	0.332
East Singhbhum	p-value		—		0.086		
Khunti	p-value			—	0.047	0.987	0.992
Lohardaga	p-value				—	0.009	0.166
Pakur	p-value					—	0.827
West Singhbum	p-value						—
Tukey Post-Hoc Test – Total Private Patient							
Deoghar	p-value	—	0.003	0.153	0.996	0.048	0.3
East Singhbhum	p-value		—		0.012		
Khunti	p-value			—	0.052	0.994	0.999
Lohardaga	p-value				—	0.014	0.119
Pakur	p-value					—	0.942
West Singhbum	p-value						—
Tukey Post-Hoc Test – Out of Expenditure per patient							
Deoghar	p-value	—	1	0.143	0.854	0.005	0.684
East Singhbhum	p-value		—	0.087	0.731	0.003	0.535
Khunti	p-value			—	0.744	0.738	0.896
Lohardaga	p-value				—	0.09	1
Pakur	p-value					—	0.171
West Singhbum	p-value						—

## Discussion

SNC-NTEP is a national exercise done to identify the districts doing well towards the elimination of TB in India. It also helps the central TB division to strategize their region-wise focus in the areas which need more attention. With this exercise, the policymakers tend to have an idea of what has worked and what needs to be improved to achieve the goal sooner.

The present study of 2022 helped us to understand the intricacies in the six studied districts of Jharkhand regarding the costs incurred upon the direct cost of antitubercular drugs. These are those districts of tribal state who claimed bronze medal i.e., >20% decrease in the incidence of TB. In our understanding, this is the first such study where the direct out-of-pocket expenses are being calculated exclusively from drug sales data of the region lending it an indigenous nature and giving a real picture. Despite the free drugs available under NTEP, some people prefer to buy drugs privately due to their own personal reasons and stigma which also need to be studied holistically in achieving the SDG target in 2030 and the ambitious target set by GOI in 2025 [3,5].

It has been noted in earlier studies that the cost incurred in TB treatment is a catastrophic health expenditure, particularly in the patients utilizing private hospitals leading to hardship financing and extra health care burden [15-18]. This holds true for drug-sensitive TB as well as MDRTB [19]. In our study findings, it has been observed that OOPE per patient due to the cost of drugs ranges from INR 743 (95% CI 372, 1113) of a least incurring cost district to INR1859 (95%CI 1728, 1990) in the highest district. In an Indian study done in 2017-2018, OOPE in patients seeking treatment from private healthcare facilities is substantially more (12100 INR vs 6800 INR) in comparison to patients seeking treatment from public health facilities leading to catastrophic costs for some families. The catastrophic costs are mainly contributed by the drugs bought from the private sector [20]. So other than the indirect costs and direct costs incurred by patients on consultation charges and diagnosis; expenses on TB drugs contribute majorly to their catastrophe.

This study estimated the yearly sales of anti-TB drugs from 2015 to 2021in six districts whose mean yearly sales varied from approximately INR 4 lakhs to 40 lakhs depending on the nature of the treatment-seeking behaviour of the patients. The costs varied in the districts due to the various combinations containing Rifampicin made available from three different companies to private stakeholders. The private practitioners had their own choice of prescribing different drug combinations available in the market as suited to them and their patients. So newer programmatic initiatives must be undertaken in these districts to decrease the catastrophic expenditure and better notification.

The total number of private patients seeking anti-TB treatment from other than the government sector has been poorly studied and there is no robust mechanism in NTEP for their documentation [21]. Recently, the program has made some provisions to track private-sector TB patients, but there is no fool-proof mechanism to capture information about all such patients. Although few researchers have estimated the disease burden based on drug sales data, private patient notification or both which showed that India’s private sector is catering to a substantial number of TB patients much higher than the earlier estimates [22-26]. The six districts studied in our research for a period of seven years (2015-2021) showed an increasing trend for the initial two years then a steady decrease in total private patients calculated from drug sales data calculated during SNC-NTEP activity in 2022. Approximately 40% of the TB patients are seeking treatment from the private sector in these districts based on NI-KSHAY portal data.

During this study, we confirmed the data on TB patients taking treatment sector from district authorities and we noticed that 5-10% of patients seeking treatment from the private sector are not being notified to the NTEP program due to varied reasons which needs to be brought under government surveillance for achieving the ambitious target of TB elimination by 2025.

Limitations

OOPE has been calculated from sales of commercially available anti-TB drugs, but ideally it should be based on all indirect and direct costs incurred during the management of TB patients. Secondly, TB patients seeking treatment from other districts may lead to an increase or decrease in sales of ATT drugs in that district, though we have tried our best to eliminate this bias by NGT and KII but it cannot be eliminated completely. Third, Schedule H1 reporting of anti-TB drugs is not complete because of which we may have missed out sales of few anti-TB drugs, thus the study findings may not depict a complete picture of the situation but undoubtedly, this will open new areas of research in TB and will help in reaching our goal of elimination by 2025.

## Conclusions

Very few studies like ours have been done till now in India where cumulative cost calculation has been done on drugs sold in the market through the state drug control directorate and not from the patients. The market of private anti-TB drugs has been vast, complicated, and sustaining for the last several decades, particularly in high-burden countries. There is a dire need for appropriate policy regarding market responses in areas of private-public partnership, greater reach of public programs, and regulatory enforcement.
